# The Relationship Between Nursing Managers’ Leadership Styles and Nurses’ Artificial Intelligence Literacy: A Cross‐Sectional Study

**DOI:** 10.1155/jonm/9246900

**Published:** 2026-04-08

**Authors:** Jiale Tong, Juan Xie, Ying Xiang, Yue Zhou, Peng Deng

**Affiliations:** ^1^ Department of Emergency Medicine, West China Hospital, Sichuan University-West China School of Nursing, Sichuan University, Wuhou District 37 Guoxue Lane, Chengdu, Sichuan, 610041, China, scu.edu.cn; ^2^ Institute of Disaster Medicine, Sichuan University, Wuhou District 37 Guoxue Lane, Chengdu, Sichuan, 610041, China, scu.edu.cn; ^3^ Nursing Key Laboratory of Sichuan Province, Wuhou District 37 Guoxue Lane, Chengdu, Sichuan, 610041, China, scu.edu.cn; ^4^ Department of Emergency Medicine, West China Hospital, and Disaster Medical Center, Sichuan University, Wuhou District 37 Guoxue Lane, Chengdu, Sichuan, 610041, China, scu.edu.cn

## Abstract

**Aim:**

To determine the relationship between nursing managers’ diverse leadership styles and nurses’ artificial intelligence (AI) literacy.

**Background:**

Nurses’ AI literacy serves as a core competency for optimizing clinical workflows and safeguarding patient safety. Although leadership has been recognized as a critical factor in the adoption of technology, empirical evidence linking specific leadership styles to AI literacy among nurses remains unclear.

**Methods:**

A cross‐sectional study was conducted between October and November 2025, involving 1644 nurses recruited from 15 general tertiary hospitals across Sichuan, Jilin, Tibet, Hunan, and Hubei Provinces in China. Data were collected using five standardized instruments: a demographic information form, the AI Literacy Scale, the Multifactor Leadership Questionnaire (MLQ), the Authentic Leadership Questionnaire (ALQ), and the Servant Leadership Questionnaire (SLQ). The univariate analysis, Pearson correlation analysis, and multiple linear regression analysis were employed to examine the relationships among the study variables.

**Results:**

Univariate analysis revealed significant differences in nurses’ AI literacy associated with work experience, received AI‐related training, and prior experience in using AI (all *p* < 0.05). Four leadership styles (transformational, transactional, authentic, and servant leadership) were observed to be positively correlated with AI literacy (*r* = 0.184–0.378, *p* < 0.001). Multiple linear regression identified five significant predictors of AI literacy: received AI‐related training (*β* = 0.147, *p* < 0.001), prior experience in using AI (*β* = 0.131, *p* < 0.001), transformational leadership (*β* = 0.163, *p* < 0.001), transactional leadership (*β* = 0.073, *p* = 0.037), and authentic leadership (*β* = 0.113, *p* = 0.015), with the model explaining 20.2% of variance.

**Conclusions:**

Our study demonstrated that transformational, transactional, and authentic leadership in nursing managers were positively associated with nurses’ AI literacy, whereas servant leadership showed no significant predictive effect. These findings highlight the multidimensional nature of leadership in fostering AI literacy among nurses and inform the strategic design of targeted leadership development initiatives within healthcare organizations.

## 1. Introduction

The advent of artificial intelligence (AI) is fueling a profound digital transformation in the healthcare sector [1]. Specifically, AI is shaping a compelling model for nursing practice, encompassing AI‐based clinical decision support, risk prediction, and intelligent robot‐assisted care [2–4]. In clinical practice, nurses increasingly encounter AI technologies across multiple domains of patient care. These include AI‐driven decision support systems embedded in electronic health records for fall risk prediction and early warning scores, intelligent monitoring systems that detect potential adverse events from real‐time vital sign data, and algorithmic tools for patient flow management and discharge planning. In some clinical settings, nurses also utilize AI‐powered documentation systems designed to streamline nursing records and reduce administrative workload. However, this transformation also introduces more serious challenges for nursing staff, such as insufficient digital literacy, risks to data security and patient privacy protection, and ethical responsibilities in the application of AI [5]. AI literacy is defined as an individual’s ability to understand, assess, and apply AI. It encompasses knowledge of its concepts, applications, ethical implications, and potential risks [6]. In this study, AI literacy was operationalized as the set of competencies necessary for effective and ethical engagement with these workplace technologies. These competencies encompass the ability to comprehend AI‐generated outputs, critically evaluate their clinical relevance, apply them appropriately in patient care decisions, and engage in reflective consideration of the ethical dimensions of AI‐assisted practice. Nurses, as the mainstay of healthcare services, are transitioning from traditional caregiving roles to complex professionals equipped with big data analysis, smart device management, and collaborative decision‐making capabilities [7]. Enhancing their AI literacy has emerged as a critical component in improving healthcare efficiency and ensuring patient safety [8].

In organizational management, various leadership styles impact team behavior via distinct pathways. Transformational leadership focuses on articulating a shared vision and unlocking subordinates’ potential, thereby effectively fostering their innovative behavior [9]. Transactional leadership involves guiding employee performance through structured reward and punishment frameworks [10]. Authentic leadership promotes psychological safety by building trust through self‐awareness, transparent communication, and ethical consistency [11]. Servant leadership concentrates on constructing supportive work environments by empowering subordinates and prioritizing employee needs [12]. Confronted with the uncertainty brought by AI, the pressure to update skills, and the anxiety over career displacement, nursing teams urgently require a leadership approach that bridges resistance, ignites motivation, and charts a clear course forward. Therefore, examining the relationship between nursing managers’ leadership styles and nurses’ AI literacy constitutes the crucial element for addressing problems in enhancing nurses’ AI literacy from an organizational management perspective.

The majority of current studies on AI in nursing consider technical feasibility and clinical application outcomes, or investigate nurses’ perceptions and attitudes toward AI [13–15]. However, no research has been conducted from an organizational management perspective examining the impact of different leadership styles on nurses’ AI literacy. Determining precisely which leadership styles more effectively facilitate the enhancement of nurses’ AI literacy could enable nursing managers to identify and develop efficient leadership models suited to the digital era. This makes it possible to create targeted management strategies and cultivate a highly skilled nursing workforce that will thrive in future smart healthcare settings. Therefore, this study aimed to identify the intrinsic relationship between different leadership styles of nursing managers and nurses’ AI literacy.

## 2. Methods

### 2.1. Study Design, Setting, and Participants

This cross‐sectional study was conducted between October and November 2025. Participants were recruited via convenience sampling from 15 general tertiary hospitals across Sichuan, Jilin, Tibet, Hunan, and Hubei Provinces in China. Inclusion criteria are as follows: (1) registered nurses holding professional licenses, (2) possession of at least one year of work experience, and (3) delivering direct clinical care for patients. Nurses who were on leave (pregnancy, illness, or personal leave) during the data collection period were excluded. In addition, nurses in managerial positions were not eligible to participate, as this study focused exclusively on the perspectives of clinical staff nurses.

A priori power analysis was performed using G∗Power software to ensure adequate statistical power. Given that the primary analytical method involved a multiple linear regression model with 13 predictor variables, the power analysis determined the minimum sample size to be 139 participants based on the following criteria: an alpha level of 0.05, an effect size of 0.15, and a statistical power of 0.8.

### 2.2. Data Collection

At each participating hospital, a designated nurse investigator was responsible for screening potential subjects and for distributing, collecting, and initially reviewing the questionnaires. Before data collection, all participants were provided with a verbal explanation outlining the research aims and importance and their right to withdraw voluntarily without penalty. Participants were instructed to access and complete the questionnaire by scanning the QR code on the Questionnaire Star platform. To ensure data integrity, we configured the platform to prevent multiple submissions from a single IP address or account and required all missing items to be completed before submission. The questionnaires with regular responses and confusing logic were excluded. A total of 1758 questionnaires were collected. All clinical nurses were asked to evaluate both the leadership styles of their respective nurse managers and their own AI literacy. After excluding invalid responses, 1644 valid questionnaires were retained, yielding an effective response rate of 93.5%.

### 2.3. Measurements

#### 2.3.1. Demographic Information Form

The structured questionnaire captured nine critical variables, including gender, age, marital status, educational background, unit of work, work experience, professional title, received AI‐related training, and prior experience in using AI.

#### 2.3.2. AI Literacy Scale

The AI Scale compiled by Wang [16] was employed to assess the AI literacy among clinical nurses. The scale encompasses four dimensions: “awareness (3 items),” “use (3 items),” “evaluation (3 items),” and “ethics (3 items).” Awareness: the ability to recognize and understand the technological nature of AI when using AI; use: the operational ability to skillfully complete specific tasks using AI; evaluation: the ability to select and critically evaluate AI applications and their outcomes; ethics: the ability to recognize relevant responsibilities and risks when using AI and to adhere to ethical guidelines. Responses were scored on a 7‐point Likert scale ranging from 1 (“*strongly disagree*”) to 7 (“*strongly agree*”), resulting in a total score range of 12–84, with higher scores indicating superior AI literacy. In this study, the scale’s Cronbach’s α was 0.786.

#### 2.3.3. Multifactor Leadership Questionnaire (MLQ)

The MLQ, originally developed by Avolio [17] and subsequently translated into Chinese by Pan [18], was utilized to measure the multifaceted leadership styles of nursing managers as perceived by nurses. This scale comprises two subscales: transformational leadership and transactional leadership. The transformational leadership subscale is composed of four dimensions, including “leadership charm (5 items),” “intellectual stimulation (5 items),” “vision motivation (5 items),” and “individualized consideration (5 items).” The transactional leadership subscale consists of three dimensions: “contingent reward (4 items),” “proactive exception management (4 items),” and “passive exception management (4 items).” The MLQ is a 5‐point Likert scale, ranging from “not at all” to “almost always,” scored from 0 to 4 points, respectively. For each subscale, higher scores indicate a stronger propensity of the nursing manager toward that particular leadership style. In the present study, the Cronbach’s α of MLQ was 0.943.

#### 2.3.4. Authentic Leadership Questionnaire (ALQ)

The ALQ, developed by Walumbwa [19] and revised by Han [20], is used to evaluate staff nurses’ perceptions of their nurse managers’ authentic leadership. The scale consists of 16 items, which correspond to four dimensions: “transparency (5 items),” “self‐awareness (4 items),” “balanced processing (3 items),” and “moral and ethical perspective (4 items).” Each item is scored from 1 (“*strongly disagree*”) to 5 (“*strongly agree*”). Higher scores indicate a stronger manifestation of the corresponding authentic leadership style in nursing managers. In this study, the Cronbach’s α of ALQ was 0.973.

#### 2.3.5. Servant Leadership Questionnaire (SLQ)

The SLQ was initially compiled by Liden [21] and translated and adapted into Chinese by Shi [22]. This scale is unidimensional and consists of 7 items. All items are rated on a 5‐point Likert scale ranging from 1 (“*extremely inconsistent*”) to 5 (“*extremely consistent”).* The total score ranges from 7 to 35, with higher scores indicating that nurses perceived their nurse managers as demonstrating higher levels of servant leadership. In this study, the Cronbach’s α of SLQ was 0.937.

### 2.4. Data Analysis

The data analyses were conducted using SPSS 27.0 software. Descriptive statistical methods (cases, frequencies, means, and standard deviations) were used to report the demographic characteristics among clinical nurses and the scores for each research variable. AI literacy scores were examined using independent‐samples *t*‐tests and one‐way analysis of variance (ANOVA) across different demographic characteristics among clinical nurses. Pearson correlation analyses were conducted to identify the relationship between various leadership styles of nursing managers and AI literacy in clinical nurses. A multiple linear regression analysis was performed to determine the independent predictive contributions of clinical nurses’ demographic characteristics and nursing managers’ leadership styles to their AI literacy. Multicollinearity among the predictor variables was evaluated using the variance inflation factor (VIF). VIF < 5 was considered to indicate the absence of significant multicollinearity. *p* < 0.05 was considered statistically significant for all two‐tailed tests.

## 3. Results

### 3.1. Participants’ Characteristics and Univariate Analysis

A total of 1644 clinical nurses were included. The majority of participants were female (93.3%) and aged 30–45 years (68.2%). Furthermore, most participants were married (72.5%) and had an undergraduate education (84.8%). In terms of work units, respondents were primarily employed in medical wards (27.9%). Approximately half (47.2%) of the sample reported having 11–20 years of work experience, and over half (50.7%) were classified at the intermediate professional title level (a mid‐level certification in the Chinese nursing career ladder requiring several years of clinical experience and passage of a provincial exam). While most nurses (65.3%) had prior experience using AI, only about one‐third (32.2%) had received AI‐related training. Detailed demographic characteristics of participants are shown in Table 1.

**TABLE 1:** Participants’ characteristics and univariate analysis (*N* = 1644).

Variables	*N* (%)	AI literacy	*t*/*F*	*p*
Gender			−0.629	0.529
Male	110 (6.7)	62.57 ± 9.90		
Female	1534 (93.3)	63.12 ± 8.80		
Age (years)			2.923	0.054
< 30	351 (21.4)	62.57 ± 7.97		
30–45	1121 (68.2)	63.43 ± 9.16		
> 45	172 (10.5)	61.92 ± 8.64		
Marital status			1.229	0.293
Unmarried	387 (23.5)	62.67 ± 8.02		
Married	1192 (72.5)	63.28 ± 9.04		
Divorced/widowed	65 (4.0)	61.98 ± 10.55		
Educational background			1.888	0.152
Junior college	231 (14.1)	62.32 ± 9.03		
Undergraduate	1394 (84.8)	63.25 ± 8.84		
Postgraduate	19 (1.2)	60.53 ± 8.83		
Unit of work			1.726	0.103
Medical ward	458 (27.9)	63.28 ± 9.29		
Surgical ward	337 (20.5)	63.69 ± 8.49		
Obstetrics and gynecology department	62 (3.8)	65.21 ± 9.26		
Pediatrics	57 (3.5)	63.39 ± 9.26		
Intensive care unit	152 (9.2)	63.28 ± 7.81		
Emergency department	213 (13.0)	62.54 ± 8.67		
Other units	365 (22.2)	62.13 ± 9.03		
Work experience (years)			3.212	0.022
≤ 5	284 (17.3)	62.33 ± 7.64		
6–10	316 (19.2)	63.99 ± 8.88		
11–20	776 (47.2)	63.35 ± 9.25		
> 20	268 (16.3)	62.06 ± 8.87		
Professional title			1.296	0.274
Junior	658 (40.0)	62.96 ± 8.54		
Intermediate	833 (50.7)	63.36 ± 9.25		
Senior	153 (9.3)	62.16 ± 8.12		
Received AI‐related training			10.357	< 0.001
Yes	529 (32.2)	66.28 ± 8.25		
No	1115 (67.8)	61.57 ± 8.76		
Prior experience in using AI			9.829	< 0.001
Yes	1073 (65.3)	64.61 ± 8.44		
No	571 (34.7)	60.22 ± 8.97		

Univariate analysis (Table 1) revealed significant differences in AI literacy among clinical nurses based on their work experience, AI‐related training, and prior experience of using AI. Specifically, nurses who had received AI‐related training demonstrated significantly higher AI literacy than those who had not. Furthermore, nurses with prior experience of using AI reported markedly higher AI literacy scores than those without such experience. In contrast, no statistically significant differences were observed in relation to gender, age, marital status, educational background, unit of work, and professional title.

### 3.2. Nurses’ AI Literacy and Perceived Leadership Styles of Their Nurse Managers

The overall mean score of the AI literacy scale among clinical nurses was 62.09 (SD = 8.87). Among all dimensions of AI literacy, participants scored highest in “awareness” (mean = 16.69, SD = 3.13), followed by “ethics” (mean = 16.64, SD = 3.04) and “evaluation” (mean = 16.16, SD = 3.25), respectively. The “use” dimension received the lowest score (mean = 13.61, SD = 2.19). As perceived by the clinical nurses, the mean scores for the nursing managers’ various leadership styles were as follows: transformational leadership (mean = 58.71, SD = 15.72), transactional leadership (mean = 25.97, SD = 5.61), authentic leadership (mean = 66.89, SD = 11.50), and servant leadership (mean = 40.80, SD = 8.60). Detailed descriptive statistics for all scales and their subscales are outlined in Table 2.

**TABLE 2:** Descriptive statistics of the AI literacy among nurses and perceived leadership styles of their nursing managers.

Variables	Min	Max	Mean ± SD
AI literacy	25	84	62.09 ± 8.87
Awareness	6	21	16.69 ± 3.13
Use	3	21	13.61 ± 2.19
Evaluation	3	21	16.16 ± 3.25
Ethics	3	21	16.64 ± 3.06
Transformational leadership	0	80	58.71 ± 15.72
Leadership charm	0	12	8.09 ± 2.43
Intellectual stimulation	0	32	24.28 ± 6.33
Vision motivation	0	20	15.28 ± 4.46
Individualized consideration	0	16	11.06 ± 3.81
Transactional leadership	9	47	25.97 ± 5.61
Contingent reward	0	15	11.19 ± 3.39
Proactive exception management	1	16	12.48 ± 3.08
Passive exception management	0	16	6.35 ± 3.25
Authentic leadership	16	80	66.89 ± 11.50
Transparency	5	25	21.06 ± 3.85
Self‐awareness	4	20	16.86 ± 3.08
Balanced processing	3	15	12.41 ± 2.24
Moral and ethical perspective	4	20	16.56 ± 2.97
Servant leadership	7	49	40.80 ± 8.60

### 3.3. Relationship Between the Nursing Managers’ Leadership Styles and Nurses’ AI Literacy

As shown in Table 3, significant positive correlations were observed between the four leadership styles among nursing managers and clinical nurses’ AI literacy and its four dimensions: “transformational leadership” (*r* = 0.213–0.378, *p* < 0.001), “transactional leadership” (*r* = 0.184–0.320, *p* < 0.001), “authentic leadership” (*r* = 0.249–0.355, *p* < 0.001), and “servant leadership” (*r* = 0.215–0.324, *p* < 0.001).

**TABLE 3:** Pearson correlation analysis results (*N* = 1644).

Variables	Transformational leadership	Transactional leadership	Authentic leadership	Servant leadership
AI literacy	0.378^∗∗^	0.320^∗∗^	0.355^∗∗^	0.324^∗∗^
Awareness	0.338^∗∗^	0.283^∗∗^	0.299^∗∗^	0.269^∗∗^
Use	0.213^∗∗^	0.235^∗∗^	0.239^∗∗^	0.215^∗∗^
Evaluation	0.292^∗∗^	0.269^∗∗^	0.286^∗∗^	0.265^∗∗^
Ethics	0.288^∗∗^	0.184^∗∗^	0.249^∗∗^	0.228^∗∗^

^∗∗^ *p* < 0.001.

The multiple linear regression results (Table 4) indicated that received AI‐related training (*β* = 0.147, *p* < 0.001), prior experience in using AI (*β* = 0.131, *p* < 0.001), transformational leadership (*β* = 0.163, *p* < 0.001), transactional leadership (*β* = 0.073, *p* < 0.05), and authentic leadership (*β* = 0.113, *p* < 0.05) were significantly correlated with nurses’ AI literacy, accounting for 20.2% of the total variance. Furthermore, no significant association was identified between servant leadership and clinical nurses’ AI literacy. The VIF values for all independent variables ranged from 1.017 to 4.851. These results confirm the absence of severe multicollinearity, thereby ensuring the stability and reliability of the regression estimates.

**TABLE 4:** Multiple linear regression analysis results (*N* = 1644).

Variables	*B*	SE	*β*	*t*	*p*	VIF
Constant	55.181	1.590	—	34.695	< 0.001	—
Work experience (ref = < 5 years)						
6–10 years	0.315	0.492	0.014	0.641	0.522	1.033
11–20 years	1.963	1.036	0.042	1.896	0.058	1.018
> 20 years	1.389	1.077	0.029	1.289	0.198	1.017
Received AI‐related training (ref = No)						
Yes	2.788	0.466	0.147	−5.986	< 0.001	1.239
Prior experience in using AI (ref = No)						
Yes	2.441	0.458	0.131	−5.326	< 0.001	1.246
Transformational leadership	0.092	0.027	0.163	3.364	< 0.001	4.851
Transactional leadership	0.116	0.055	0.073	2.089	0.037	2.521
Authentic leadership	0.087	0.036	0.113	2.433	0.015	4.428
Servant leadership	0.035	0.044	0.034	0.811	0.148	3.704

*Note:* B, the unstandardized coefficient; β, the standardized coefficient; SE, the standard error; *R* ^2^ = 0.206; adjusted *R* ^2^ = 0.202.

## 4. Discussion

This study was conducted to determine the relationship between clinical nursing managers’ leadership styles and clinical nurses’ AI literacy. Our findings demonstrated that transformational, transactional, and authentic leadership were positively associated with AI literacy in nurses. These findings highlight the strategic importance of leadership development for enhancing nurses’ AI literacy, thus providing an evidence‐based impetus for nursing managers to refine their practices and furnishing healthcare institutions with a clear rationale for designing targeted training programs.

Transformational leadership is a process in which leaders transform subordinates’ values and attitudes through their personal charisma and inspiration [23]. It generally comprises four critical components: “leadership charm,” “intellectual stimulation,” “vision motivation,” and “individualized consideration [24].” Our findings indicated that transformational leadership was positively correlated with clinical nurses’ AI literacy. Previous studies have demonstrated that transformational leaders foster in subordinates a commitment to collective interests over self‐interest by enabling them to identify with a shared vision and appreciate the importance of their tasks [25, 26]. Improving patient health outcomes and organizational advancement are universal aspirations shared by clinical nurses and nursing administrators. While AI emerges as a transformative technology with significant potential to enhance nursing efficiency and improve patient safety, its inherent complexity and the ethical concerns it raises pose substantial challenges for nurses in daily practice [27]. Transformational leaders, generally characterized by their advocacy for innovation, proactively assume the risks associated with AI implementation [28]. By serving as role models and actively devising strategies to overcome AI‐related challenges, they foster a climate of psychological safety within the nursing team. This leadership style is instrumental in positively reshaping nurses’ perceptions and attitudes toward AI.

Transactional leadership is a leadership style founded on explicit exchanges, wherein the leader motivates subordinates toward organizational objectives by clearly defining their roles and by establishing a structured system of rewards and punishments [29]. Our findings revealed that transactional leadership was positively associated with nurses’ AI literacy. According to Social Exchange Theory, interpersonal interaction constitutes an implicit process of exchange governed by the norm of reciprocity [30]. In other words, individuals who benefit from the actions of others or an organization are expected to reciprocate in some way. Transactional leadership establishes clear role definitions and a structured framework of rewards and sanctions, creating explicit conditions for the exchange of services between nurses and their leaders [31]. Nurses generally encounter AI technologies in various aspects of their daily clinical practice. These include AI‐driven clinical decision support systems that provide fall risk predictions, intelligent early warning systems that detect patient deterioration, and algorithms embedded in electronic health records that assist with patient flow management and documentation. These tools require nurses to actively engage with AI‐generated outputs, interpret their clinical significance, and integrate them into patient care decisions. When nurses receive recognition, rewards, or career development opportunities from their leaders for demonstrating proficiency in using these AI tools, they are more likely to engage in learning and practicing AI‐related skills, as they develop a sense of obligation rooted in the norm of reciprocity. For instance, a nurse whose manager publicly acknowledges their correct interpretation of an AI‐generated fall risk alert may feel motivated to further develop their competency in using such systems. Over time, these behaviors become internalized within the organizational context and gradually manifest as enhanced AI literacy.

Authentic leadership is defined as a leadership behavior pattern characterized by the sustenance of self‐awareness, an internalized moral perspective, balanced information processing, and relational transparency, all built upon a foundation of positive psychological capacities and an ethical climate to enable self‐development [32]. In our study, authentic leadership was observed to have a significant positive correlation with nurses’ AI literacy. Self‐determination theory posits that the fulfillment of an individual’s intrinsic motivation is dependent upon the satisfaction of three basic psychological needs, which are autonomy, competence, and relatedness [33]. Authentic leadership cultivates nurses’ intrinsic motivation to engage in AI learning by satisfying their basic psychological needs. First, authentic leaders demonstrate relational transparency and balanced processing, supporting nurses’ autonomy and respecting their input during the application of AI. Second, consistent support and encouragement enable nurses to grow in confidence through positive experiences in a psychologically safe environment. Furthermore, by establishing trusting and genuine relationships, they foster nurses’ sense of belonging, making them feel valued within the team. When these needs for autonomy, competence, and relatedness are fulfilled, nurses’ motivation shifts from being externally driven to being intrinsically motivated, leading to a more profound and sustainable improvement in AI literacy.

Notably, our findings showed no significant correlation between servant leadership and nurses’ AI literacy. While servant leadership brings core strengths in enhancing employees’ sense of belonging and engagement, its effectiveness in driving the adoption of specific, complex new technologies may be less direct and efficient than leadership styles that deliver clearer technical visions and structured incentives [34]. In addition, another possible explanation is that during the early stages of AI technology adoption, nurses may have refrained from seeking assistance due to anxiety or unfamiliarity, resulting in underutilization of the support resources offered by servant leadership. These findings also imply that the impact of servant leadership on AI literacy may exhibit a lag effect, with its value becoming more pronounced during the advanced application phase after nurses have mastered foundational skills.

### 4.1. Limitations

Some limitations should be considered. First, although the cross‐sectional design employed in this study identified associations among the variables of interest, it did not permit validation of causal relationships among them. It is recommended that longitudinal studies be conducted in the future to generate more robust evidence for causal inference. Second, the convenience sampling method and the settings and cultural contexts in which this research was conducted limit the generalizability of the findings. Third, the assessment of all variables in this study was based exclusively on self‐reported data, which might introduce recall bias.

### 4.2. Implications to Nursing Management

Our findings provide empirical evidence confirming the positive relationships of transformational, transactional, and authentic leadership with nurses’ AI literacy. Therefore, healthcare organizations should develop integrated leadership programs that cultivate a complementary suite of leadership styles. These initiatives should train nursing managers to articulate an inspiring vision for AI integration, for example, by highlighting how AI‐driven early warning systems can help reduce preventable patient deterioration and improve survival rates. Managers should also establish clear performance expectations and reward structures for AI skill acquisition, such as incorporating competency in using clinical decision support systems into annual performance reviews and offering continuing education credits for completing AI‐related training. Furthermore, nursing managers should foster a psychologically safe, ethical climate through transparency and trust, for instance, by encouraging nurses to voice concerns about AI‐generated recommendations and creating regular forums to discuss the ethical implications of AI‐assisted decision‐making. Adopting this multifaceted leadership strategy enables nursing managers to address structural barriers to technology adoption more effectively. This approach accelerates the development of a digitally fluent nursing workforce that can use AI to improve patient care outcomes.

## 5. Conclusions

In conclusion, this study revealed significant positive associations between clinical nurses’ perceptions of transformational, transactional, and authentic leadership in their nurse managers and the nurses’ self‐reported AI literacy. However, servant leadership was not identified as a significant predictor in this context. These findings suggest that the leadership environment, as perceived by staff nurses, may play a role in shaping their AI literacy. Given that the leadership variables accounted for a modest proportion of the variance in AI literacy (20.2%), future research should explore additional individual and organizational factors that may influence nurses’ AI competencies, such as organizational support, access to technology, and quality of training. Nevertheless, this study provides preliminary evidence for healthcare organizations to consider incorporating leadership development, particularly in transformational, transactional, and authentic leadership, as part of a multifaceted strategy to enhance nurses’ readiness for AI integration.

## Author Contributions

Jiale Tong: conceptualization, methodology, validation, formal analysis, visualization, writing−original draft, and writing−review and editing. Juan Xie: methodology and formal analysis. Ying Xiang: validation, writing−review and editing, and supervision. Yue Zhou: conceptualization, methodology, validation, writing−review and editing, and supervision. Peng Deng: conceptualization, methodology, validation, writing−review and editing, and supervision.

## Funding

The authors received no specific funding for this work.

## Ethics Statement

This study received ethical approval from the Ethics Committee of West China Hospital, Sichuan University (No. 20231846).

## Conflicts of Interest

The authors declare no conflicts of interest.

## Data Availability

The datasets used during the current study are available from the corresponding author upon reasonable request.
